# Preliminary insights into the impact of primary radiochemotherapy on the salivary microbiome in head and neck squamous cell carcinoma

**DOI:** 10.1038/s41598-020-73515-0

**Published:** 2020-10-06

**Authors:** Christina Kumpitsch, Christine Moissl-Eichinger, Jakob Pock, Dietmar Thurnher, Axel Wolf

**Affiliations:** 1 Diagnostic and Research Institute of Hygiene, Microbiology and Environmental Medicine, Medical University of Graz, Graz, Austria; 2 BioTechMed, Graz, Austria; 3 Department of Otorhinolaryngology, Medical University of Graz, Graz, Austria

**Keywords:** Chemotherapy, Radiotherapy, Microbiome, Head and neck cancer

## Abstract

Squamous cell carcinoma is the most common type of throat cancer. Treatment options comprise surgery, radiotherapy, and/or chemo(immuno)therapy. The salivary microbiome is shaped by the disease, and likely by the treatment, resulting in side effects caused by chemoradiation that severely impair patients’ well-being. High-throughput amplicon sequencing of the 16S rRNA gene provides an opportunity to investigate changes in the salivary microbiome in health and disease. In this preliminary study, we investigated alterations in the bacterial, fungal, and archaeal components of the salivary microbiome between healthy subjects and patients with head and neck squamous cell carcinoma before and close to the end point of chemoradiation (“after”). We enrolled 31 patients and 11 healthy controls, with 11 patients providing samples both before and after chemoradiation. Analysis revealed an effect on the bacterial and fungal microbiome, with a partial antagonistic reaction but no effects on the archaeal microbial community. Specifically, we observed an individual increase in *Candida* signatures following chemoradiation, whereas the overall diversity of the microbial and fungal signatures decreased significantly after therapy. Thus, our study indicates that the patient microbiome reacts individually to chemoradiation but has potential for future optimization of disease diagnostics and personalized treatments.

## Introduction

Approximately 2.9% of newly diagnosed cancers originate in the oral cavity and pharynx^1,2^. Major risk factors are tobacco and alcohol consumption, as well as infection with human papilloma virus (HPV)^2–4^. Squamous cell carcinoma (SCC), the most common type of throat cancer, is a malignant epithelial tumor that originates in the lining of the upper respiratory tract. SCC is diagnosed by a combination of clinical, radiological, and histopathological examinations, and standard treatments comprise surgery (i.e. removal of the diseased tissue), radiotherapy, and/or chemo(immuno)therapy^2^.

Radiotherapy in combination with platinum-based chemotherapy can be indicated in both surgically resectable and non-resectable SCCs. In surgically resectable cases, the literature shows a mortality benefit from adjuvant radiochemotherapy (RCHT) in patients with advanced head and neck cancer^5^. Resection techniques as well as microvascular reconstruction of the tumor side often result in large cosmetic and functional defects, and total tumor resection may not be achieved^6^. Thus, the treatment of choice in locally advanced, non-resectable cases is usually primary radiotherapy in combination with chemo(immuno)therapy.

In principle, radiotherapy and chemotherapy act by interfering with cell division, which particularly affects cancer cells that have a higher proliferation rate than healthy tissue^7,8^. Due to the non-specificity for SCC cells, local radiotherapy and/or chemo(immuno)therapy results in several side effects affecting the pharynx area. Although intensity-modulated radiotherapy treatment regimens were introduced in the early 2000s, exposure of the head and neck region to radiation still leads to severe side effects, including xerostomia, mucositis, pain and swallowing dysfunction^9^, and decreased quality of life.

More than 90% of head and neck cancer patients receiving radiotherapy develop oral mucositis, leading to hindering symptoms in approximately 40% of these patients^7,8,10^*.* Primary symptoms of oral mucositis include erythema and pain approximately 4–5 days after the start of therapy and can lead to further phases of mucositis, including local ulceration. These changes have an impact on the local environment, including microbial changes in bacteria, fungi, and viruses^10^.

Like other surfaces of the human body, the upper respiratory tract is covered in human-associated microorganisms, the microbiome^11^. Imbalances in the microbiome (dysbioses) have been linked to disease, including inflammatory bowel disease, diabetes, colon cancer, obesity, and mental illnesses. The microbiome of the upper respiratory tract comprises more than 1000 different microorganisms, including bacteria, archaea, and eukaryotes (mainly fungi). In general, the microbiome of this area is important for the integrity of the mucosa and modulation of the immune system, as human commensal bacteria have immunomodulatory properties and are able to ensure an efficient and rapid defense against pathogens by priming their host’s immune response. In addition to immune system modulation, commensal bacteria occupy the mucosa, preventing pathogen colonization due to uncontrolled settling in open niches^11^. *Streptococcus, Veillonella, Gemella, Rothia, Fusobacterium,* and *Neisseria* species are amongst the most abundant microorganisms of the upper respiratory tract; however, diseases of the upper respiratory tract, as also pharyngeal SCC, have been linked to changes in the relative abundance and overall microbial composition^11–14^.

Cancer therapy-induced oral mucositis is considered to have a microbiome component, as disruption of the mucosal barrier (caused by inflammation and apoptosis leads to bacterial translocation and an increase in the inflammatory response^15,16^. Notably, the oral microbiome could play a role in exacerbating or protecting from mucosal injury, and the roles may fluctuate depending on the stage of inflammation^15^.

Although a better understanding of the impact of radiochemotherapy (RCHT) on the oral microbiome may provide information regarding the pathophysiology and potential adjuvant treatment approaches in RCHT-induced mucositis in patients with head and neck SCC, the available literature in this field is sparse and largely restricted to cultivation-based analyses^17,18^. One exception is the study by Hu et al. in 2013^19^, in which they performed a pyrosequencing-based microbiome analysis of plaque samples taken from subjects undergoing head-and-neck radiotherapy. As high-throughput sequencing techniques and methodological strategies (e.g., selection of primers) have made substantial progress over the last few years, we think it is necessary to perform additional pilot studies for comparative reasons as a baseline for subsequent, deeper analyses^19^. Furthermore, additional studies based on high-throughput next generation sequencing of well-defined patient samples are necessary to define potential microbial processes and provide potential targets for the prevention, diagnosis, and treatment of RCHT-associated side effects.

The oral cavity microbiome has been the focus of research in relation to several diseases, including periodontitis and oral cancer^20^. For our studies, we chose to analyze the salivary microbiome to assess the local impact of radiotherapy on the overall oral microbiome using an easy, standardized, non-invasive sampling procedure that would also be suitable for screening patients^12^.

Based on the information given above, we assume (1) that interrogation of salivary microbial biomarkers could be informative with respect to the pathobiology of RCHT-related side effects (2); that the salivary, bacterial, archaeal, and fungal microbiome reflects the disease status in head and neck SCC when compared to healthy controls; and (3) that RCHT has a significant impact on the local microbiome profile. We addressed these questions in this preliminary study.

## Results

### Description of the patient cohort and study overview

For this study we recruited 42 participants (whose metadata information are given in Table 1, respectively). For a short overview see Supplementary Table S1. Parts of the cohort were recruited during our previous study (see Supplementary Table S1)^12^.

**Table 1:** Characteristics of the study participants.

Group	N	Mean age ± SD, years	Sex	HPV	Alcohol	Smoking	Tumor Localization
Healthy	11	47.8 ± 15.2	m (n = 10) f (n = 1)	n.a. (n = 11)	Never (n = 2), Occasional (n = 9)	No (n = 10) Yes (n = 1)	n.a. (n = 11)
Diseased	31 (20 + 11)	60 ± 7.5	m (n = 26) f (n = 5)	Positive (n = 7) Negative (n = 24)	Never (n = 7), Occasional (n = 14), Daily (n = 8), n.a. (n = 2)	No (n = 19) Yes (n = 12)	Oropharynx (n = 18), Hypopharynx (n = 3), Epipharynx (n = 1), Larynx (n = 3), CUP (n = 1), Oral cavity (n = 5)
Before (patients sampled only before therapy)	20	61.1 ± 7.4	m (n = 16) f (n = 4)	Positive (n = 5) Negative (n = 15)	Never (n = 4), Occasional (n = 7), Daily (n = 7), n.a. (n = 2)	No (n = 11) Yes (n = 9)	Oropharynx (n = 11), Hypopharynx (n = 3), Epipharynx (n = 0), Larynx (n = 1), CUP (n = 1), Oral cavity (n = 5)
After (patients sampled before and after therapy)	11	58 ± 7.6	m (n = 10) f (n = 1)	Positive (n = 2) Negative (n = 9)	Never (n = 3), Occasional (n = 7), Daily (n = 1)	No (n = 8) Yes (n = 3)	Oropharynx (n = 7) Hypopharynx (n = 0) Epipharynx (n = 1) Larynx (n = 2) CUP (n = 1) Oral cavity (n = 0)
All participants	42	56.8 ± 11.3	m (n = 36) f (n = 6)	Positive (n = 7) Negative (n = 24) n.a. (n = 11)	Never (n = 9), Occasional (n = 23), Daily (n = 8), n.a. (n = 2)	No (n = 29) Yes (n = 13)	Oropharynx (n = 18) Hypopharynx (n = 3) Epipharynx (n = 1) Larynx (n = 3) CUP (n = 1) Oral cavity (n = 5)

*diseased* squamous cell carcinoma patients, *m* male, *f* female, *SD* standard deviation, *CUP* carcinoma of unknown primary, *n.a.* not available.

### Description of the microbiome data

The DNA extracted from the saliva samples was subjected to three different amplification protocols: one targeting the overall microbiome (“universal” approach), one targeting the archaeal components (“archaeal” approach), and one directed to determine the fungal diversity (“fungal” approach). All sequences were quality filtered, contamination-controlled, and processed as described in the “Materials and methods”.

Following the “universal” approach, 3,339,965 sequences were obtained and classified into 23 phyla, 231 genera, and 1876 unique ribosomal sequence variants (RSVs), whereas the “fungal” approach (251,488 sequences) resulted in 5 phyla, 100 genera, and 464 unique RSVs. Possibly due to the underrepresentation of archaea in human saliva, the amplification of archaeal signals proved very difficult, and the “archaeal” approach delivered only 36,856 sequences affiliated with 4 phyla and 3 genera (all RSV tables are given in Supplementary Datasets 1, 2, and 3).

### Impact of SCC and radiotherapy on the bacterial microbial community

#### Squamous cell carcinoma affected specific taxa compared to healthy controls but did not change the overall microbiome profile

In an initial step, we re-analyzed the properties of the tumor-associated saliva microbiome by comparing the samples from healthy patients (n = 11) and SCC patients (samples from before RCHT only; n = 31)^12^. The “universal” approach identified Firmicutes as the predominant phylum, with genera *Veillonella*, *Rothia*, *Haemophilus*, and *Neisseria* being highly abundant in both healthy subjects and patients (see Supplementary Fig. S1 and S2).

**Figure 1:**
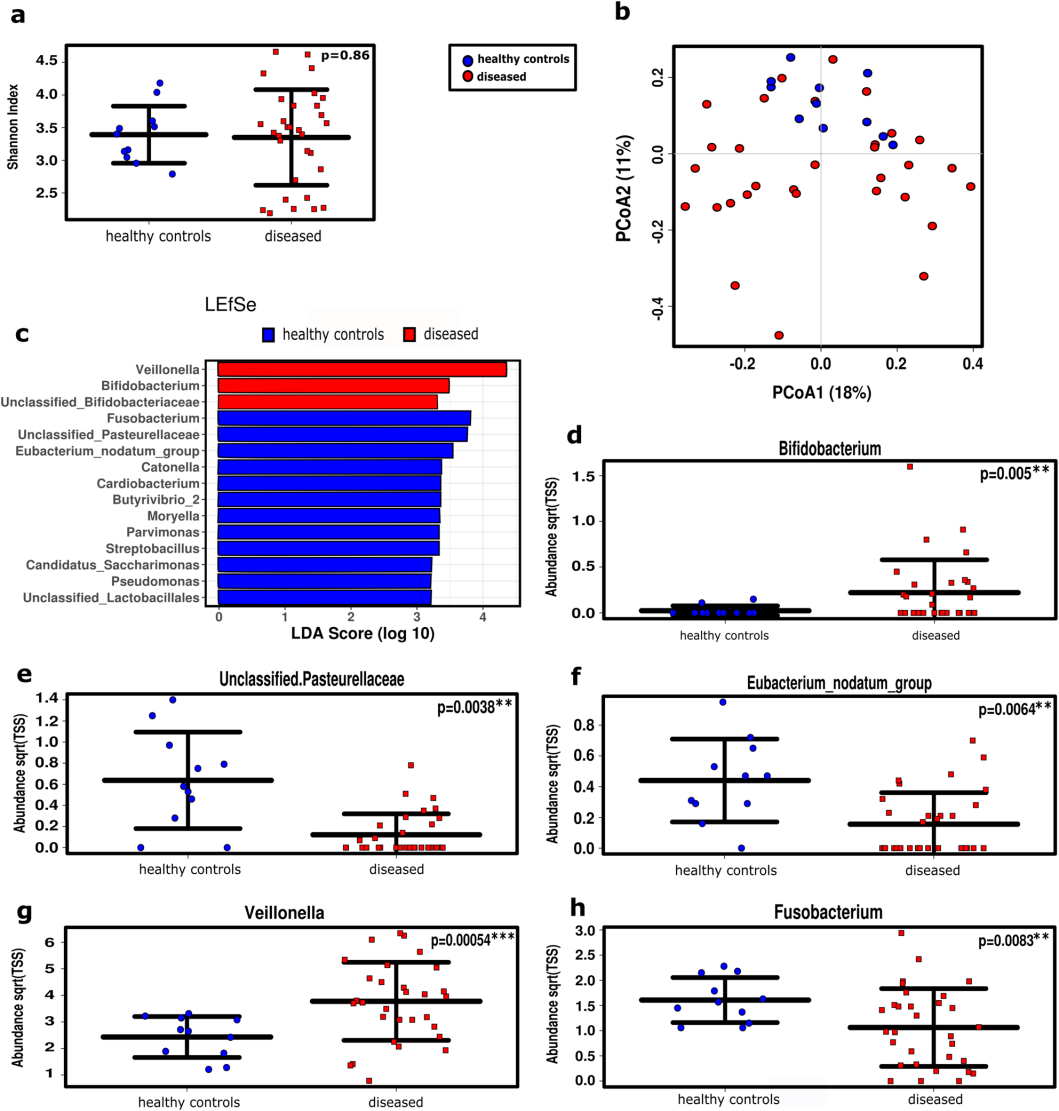
Microbial diversity and profile in cancer patients (‘diseased’) did not differ significantly from healthy controls, but healthy individuals formed a sub-cluster within the diseased cohort. (**a**) No alterations were observed in alpha diversity between the two groups at the RSV level using ANOVA. (**b**) Microbiome profile of healthy controls clustered within the diseased subjects in a PCoA plot (RSV level). (**c**) LEfSe analysis of taxa associated with healthy subjects and SCC, including the top 100 most abundant genera. (**d**–**h**) Relative abundance of the genera *Bifidobacterium*, *Pasteurellaceae*, *Eubacterium*, *Veillonella,* and *Fusobacterium* in healthy subjects compared to patients.

In congruence with our previous study^12^, we detected no significant difference in the alpha diversity between healthy controls and cancer patients (p = 0.86, ANOVA; Fig. 1a). The microbial communities of healthy controls formed a sub-cluster within the principal coordinate analysis (PCoA) plot but did not cluster separately from patient samples (Fig. 1b). Linear discriminant analysis effect size (LEfSE) analysis run at the genus level identified a potential association of *Veillonella* and Bifidobacteriaceae (including genus *Bifidobacterium*) with patients, and Pasteurellaceae, *Eubacterium* and others with healthy controls (Fig. 1c–f). *Veillonella* was significantly increased in samples from healthy subjects, which is in agreement with our previous data. Notably, *Fusobacterium* also significantly correlated with the healthy status (Fig. 1g,h), although *Fusobacterium* is increasingly being recognized as being linked to cancer development^21,22^.

#### RCHT has an impact on microbiome composition

Next, we focused on analyzing the impact of RCHT on the salivary microbiome in patients for whom we had samples from before and after RCHT (n = 11). All patients underwent additional antibiotic/mycobiotic therapy along with the RCHT, which was highly individual and adapted according to clinical requirements as shown in Supplementary Table S2 and Supplementary Fig. S3.

**Figure 2:**
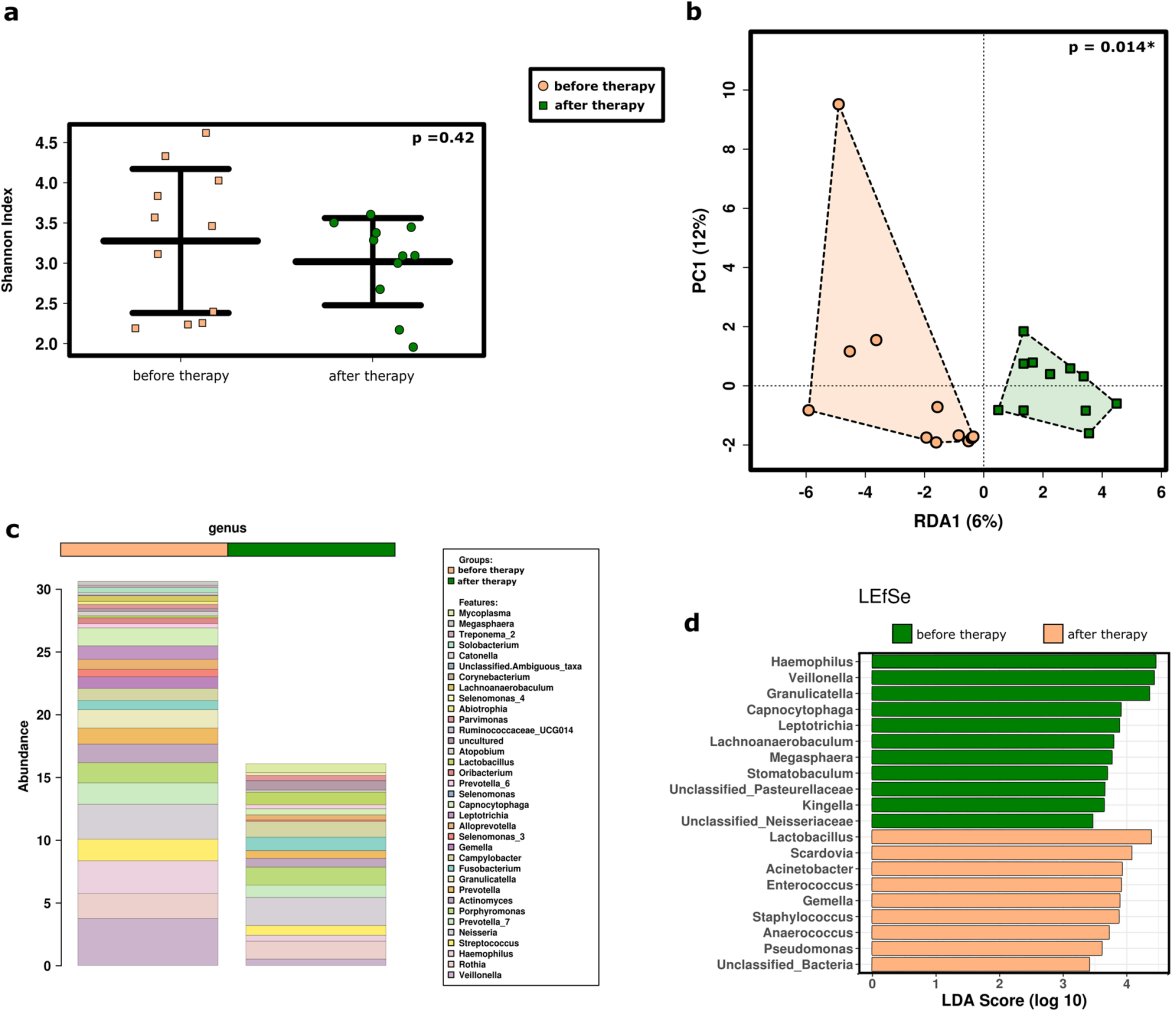
Radiochemotherapy in cancer patients substantially impacts the microbial profile. (**a**) Shannon Diversity Index did not reveal significant differences in the salivary microbiome between patients before and after therapy at the RSV level using ANOVA. (**b**) Treatment had a significant impact on the microbiome profile (RDA plot, RSV level). (**c**) Bar plot of the microbiome composition before and after therapy, including the 35 most abundant genera. Taxa are displayed from bottom to top. (**d**) Specific taxa that were significantly increased before or after therapy are plotted in a LEfSe including the 35 most abundant genera.

With the universal approach, the diversity of the microbiome decreased after therapy, but it was not significant (p = 0.42, ANOVA; Fig. 2a). According to the redundancy analysis (RDA), RCHT had a significant impact on the microbiome profile (p = 0.014; Fig. 2b), and the sample types grouped somewhat differently in the PCoA plot (see Supplementary Fig. S4). The different microbial profile as well as the difference in bacterial load were also visible in the bar-plot analysis (Fig. 2c), as the relative abundance of major taxa was significantly reduced. Overall, the derived number of sequences decreased from an average 55,092 reads per sample before RCHT to an average 38,980 reads per sample after RCHT.

We observed that some taxa were specifically affected by the treatments, such as *Haemophilus, Veillonella, Granulicatella,* and others, whereas other bacterial genera were specifically increased after treatment (*Lactobacillus, Scardovia, Acinetobacter, Enterococcus* etc.; Fig. 2d).

For the sake of completeness, we were able to confirm the correlation of alcohol consumption with the microbial profile (p = 0.075; see Supplementary Fig. S5)^12^.

### The impact of SCC and radiotherapy on the non-bacterial microbial community

#### SCC does not significantly affect the archaeal and fungal profiles

As the bacteria-centric “universal” approach is not capable of assessing the full microbial diversity and patients frequently suffer from fungi-driven mucositis, we performed additional archaeal and fungi-targeting amplicon sequencing with specific primer sets for these taxa. The archaeal dataset did not prove indicative for any of the parameters, particularly as only a small proportion of all samples revealed positive archaeal signals (20 out of 53); however, these were mainly samples from cancer patients (3 healthy controls, 13 patients before therapy, and 4 after therapy). In agreement with our previous study^12^, we confirmed the presence of *Methanobrevibacter* signatures, and detected *Methanosphaera*, and *Candidatus* Nitrosoarchaeum sequences (Supplementary Dataset 1).

**Figure 3:**
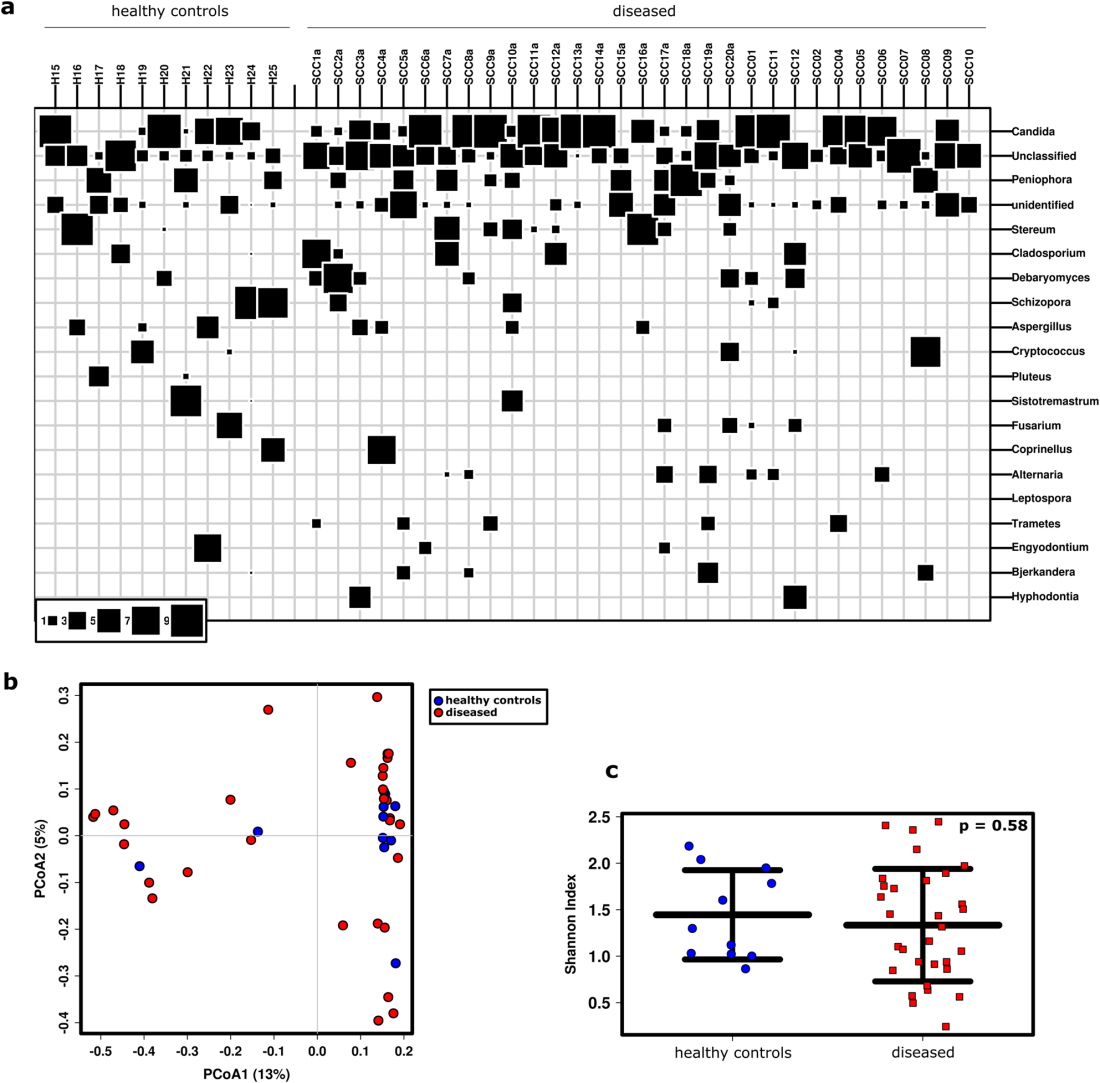
The salivary mycobiome was not significantly affected by SCC. (**a**) Bubble-plot including the top 35 most abundant genera of the study cohort [i.e., healthy controls and SCC patients before any therapy (‘diseased’)]. (**b**) PCoA plot indicating no clustering of samples from healthy subjects and diseased subjects at the RSV level. (**c**) Similar Shannon Diversity Indices in both subject groups.

Overall, 100 fungal genera were detected, with *Candida* being the most prevalent in most samples. Other frequent fungal signatures were from unclassified genera, *Peniophora, Stereum, Cladosporium,* and many others (Fig. 3a), some of which may be associated with the environment and not the human microbiome. The salivary mycobiome of healthy volunteers and SCC patients before therapy did not group differently in the PCoA plot, and no alteration in alpha diversity was observed (Fig. 3b,c).

#### RCHT does affect the archaeal and fungal profiles of salivary samples

**Figure 4:**
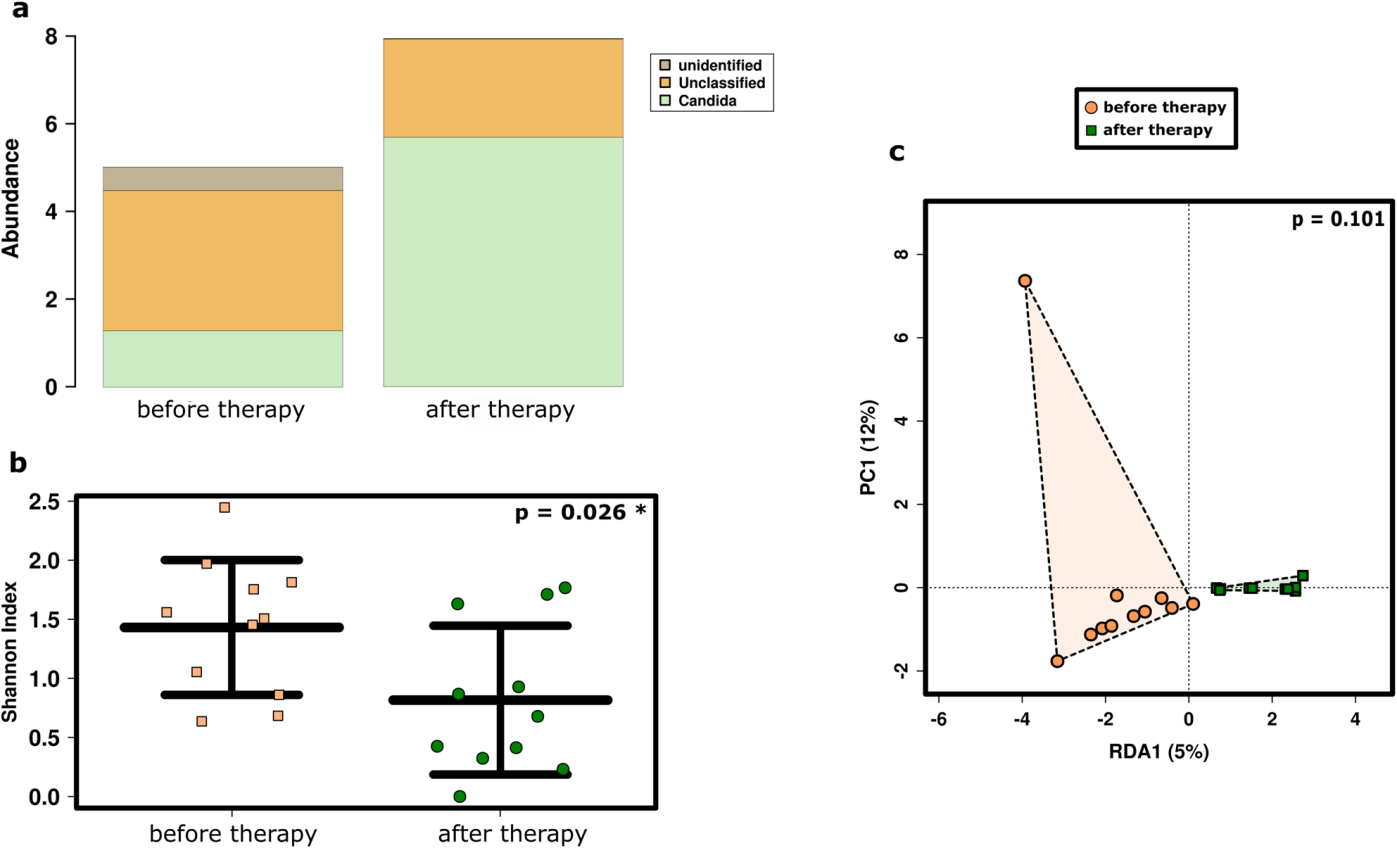
Radiochemotherapy influenced the salivary mycobiome of SCC patients. (**a**) Bar plot of the 35 most abundant fungal genera detected in patients’ saliva before and after therapy. (**b**) Alpha diversity was significantly decreased after SCC therapy at the RSV level using ANOVA. (**c**) Fungal profiles were not significantly influenced by the therapy. RDA plot; RSV level; ANOVA.

The overall relative abundance of the 35 most abundant fungal signatures increased after RCHT compared to the samples from patients before RCHT. However, the increase in relative abundance was accompanied by a significant decrease in the fungal diversity (p = 0.026; Fig. 4a,b; see also Supplementary Fig. S6).

When comparing the fungal profile of salivary samples from patients before and after RCHT, RDA indicated a potential impact of the treatment on the fungal microbial composition (p = 0.101; Fig. 4c).

**Figure 5:**
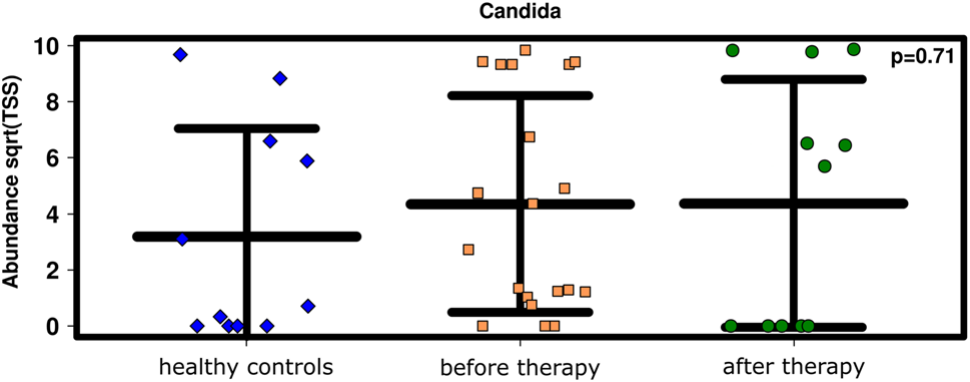
A non-significant but slight increase in the relative abundance of *Candida* was observed in patients before and after therapy compared to healthy individuals. Analysis was performed with ANOVA.

Interestingly, compared to healthy controls, the relative abundance of *Candida* was increased in patients (p = 0.39; Fig. 5). After RCHT, the relative abundance of *Candida* increased further or decreased, resulting in a total increase in the summed relative abundance (all samples; Fig. 4a). However, due to individual development for each patient, the mean relative abundance of *Candida* remained similar before and after RCHT (Figs. 4a, 5).

**Figure 6:**
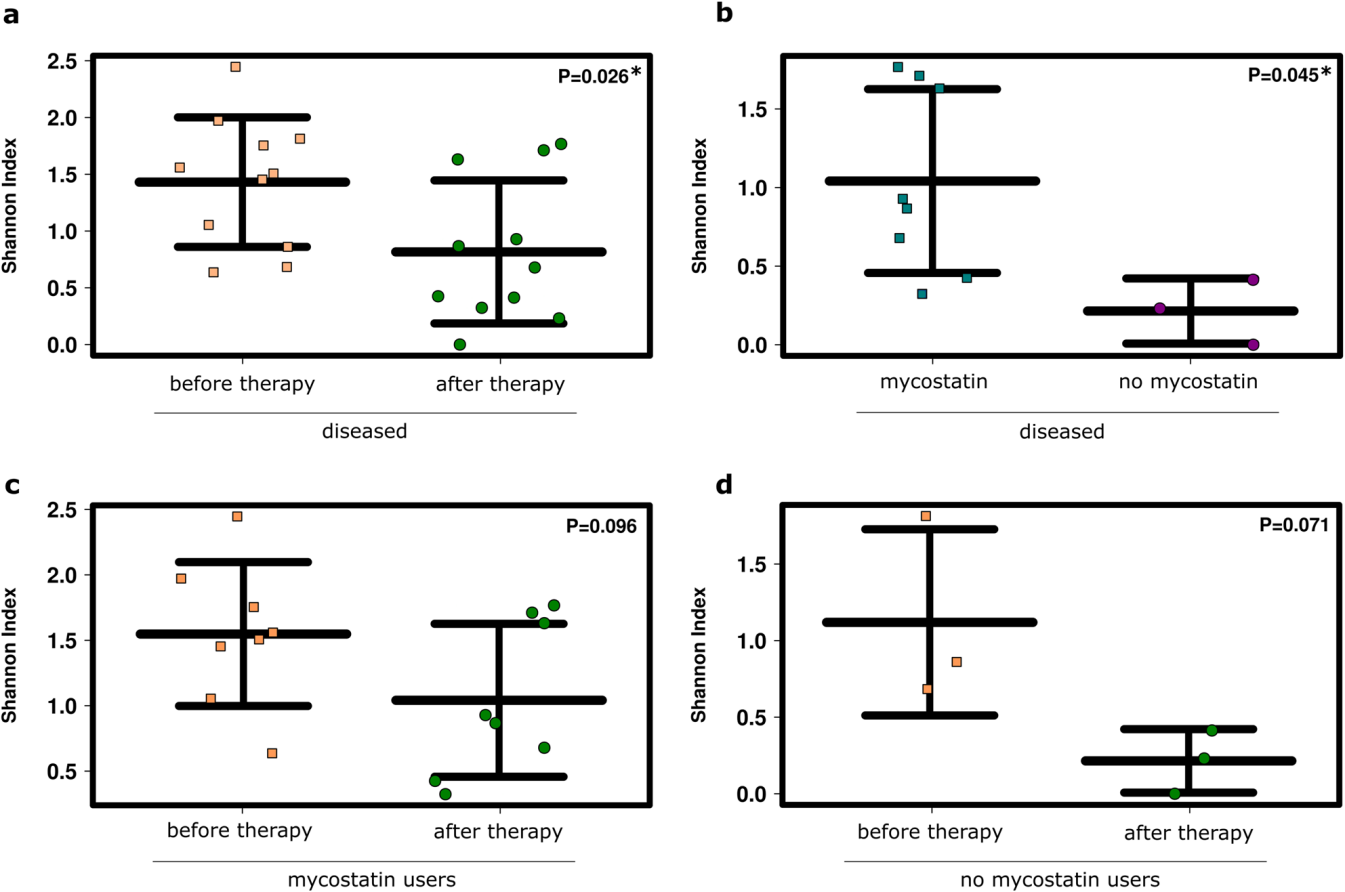
Radiochemotherapy (RCHT) and treatment with mycostatin affected the mycobiome of SCC patients. Shannon Diversity Indices of (**a**) patients before and after RCHT, (**b**) patients who did and did not receive mycostatin treatment, (**c**) mycostatin users only before and after RCHT, and (**d**) patients who did not receive any mycostatin treatment before and after RCHT. ANOVA; diseased: SCC patients.

Overall, we detected no influence of mycostatin on the fungal profile (RDA p = 0.924; patients after therapy; see also Supplementary Fig. S7), which may be due to the prevalence of environmentally associated fungal signatures in our dataset. Comparing the fungal communities of all patients (n = 11) before and after therapy, the alpha diversity decreased significantly after treatment (p = 0.026; Fig. 6a). Samples from mycostatin users revealed significantly higher fungal diversity compared to patients who did not receive mycostatin (p = 0.045; Fig. 6b).

When we evaluated the mycobiomes of mycostatin users (n = 8) before and after RCHT, a tendency towards reduced fungal diversity was still visible, but no longer significant (p = 0.096; Fig. 6c). Similarly, the alpha diversity of samples from non-users decreased after therapy (p = 0.071; Fig. 6d). As a decrease in fungal diversity was observed independent from mycostatin usage, RCHT seemed to be a major factor influencing the fungal microbiome.

Interestingly, although mycostatin is an antimycotic used to treat yeast infections, *Candida* seemed to be unaffected (see Supplementary Fig. S8a,b).

### Comparison of the microbiome and mycobiome of healthy controls with patients before and after standard therapy

**Figure 7:**
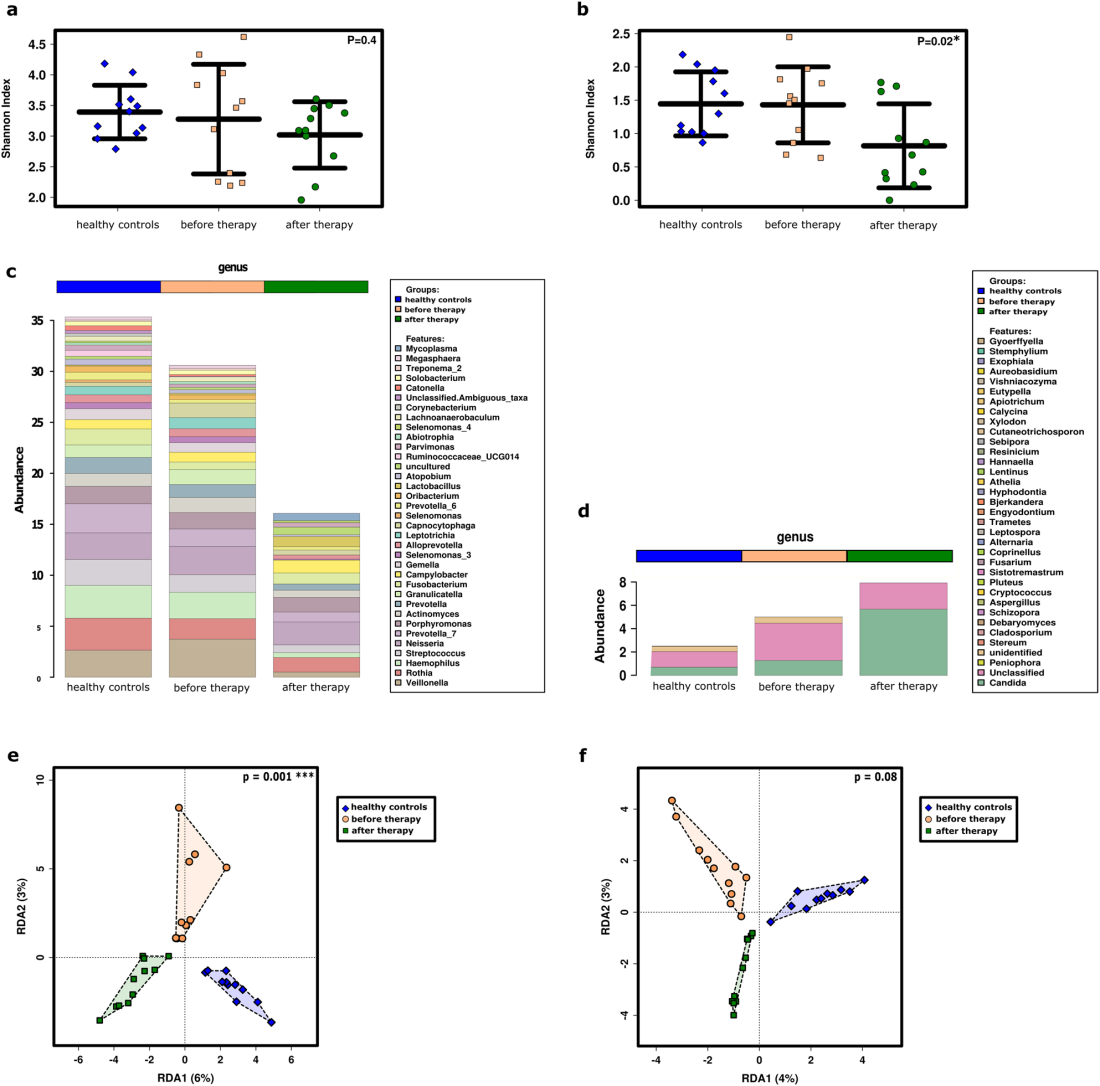
The bacterial and fungal microbiome of healthy subjects and SCC patients is more similar before radiochemotherapy than after. Shannon Diversity of the (**a**) bacterial and (**b**) fungal communities at RSV level (ANOVA). Bar plot of the top 35 most abundant (**c**) bacterial and (**d**) fungal genera. Redundancy analysis of the (**e**) bacterial and (**f**) fungal profiles using ANOVA. Taxa listed in the bar-plot are displayed from bottom to top.

The impact of disease and radiotherapy on the fungal and bacterial microbiome was specifically addressed by comparing salivary profiles from healthy controls (n = 11), SCC patients before radiotherapy (n = 11), and the same SCC patients after radiotherapy (n = 11). Overall, the bacteriome and mycobiome revealed a partially antagonistic behavior. Though the diversities of both taxonomic groups, as well as the relative abundance of bacteria, decreased from healthy to non-treated, and then to treated, patients (Shannon index, p = 0.4 (bacteria) and p = 0.02 (fungi); Fig. 7a,b), the relative abundance of fungal signatures (particularly *Candida*) increased with treatment (Fig. 7c,d, bar plots) and remained unaffected from mycostatic treatment (p = 0.924, RDA; see Supplementary Fig. S8). Notably, redundancy analyses indicated a significant impact of health and treatment status on the bacteriome, and a tendency was seen in the mycobiome (p = 0.001 and p = 0.08, respectively, RDA plots; Fig. 7e,f).

## Discussion

This preliminary study aimed for an initial comparative analysis of oral microbial communities using saliva from head and neck cancer patients and healthy controls. We found evidence that the occurrence of SCC and RCHT alone or in combination with local antimycotic treatment have a substantial impact on the microbial profile. These findings support theories that head and neck SCC itself, but also tumor-specific therapies, interacts with the oropharyngeal saliva microbiome.

The ecology of the saliva has been investigated previously in numerous studies primarily using culture-based methods^23–29^. For example, Almståhl, et al. reported the association of cultivable *Candida albicans* and enterococci with radiation-induced hyposalivation, and the presence of *Lactobacillus* spp. in 92% of all radiotherapy patients^17^. The prevalence of *Candida*, as well as enterococci and various Enterobacteriaceae (*Enterobacter, Klebsiella, Proteus, Pseudomonas*), was confirmed in a cultivation-based study focusing on radiotherapy patients^18^. Notably, lactobacilli and *Acinetobacter* have been shown to occupy open niches that can also be found after RCHT^30^.

Advances in DNA sequencing have given us the opportunity to analyze microbes that are not amenable to cultivation using high-throughput sequencing of the microbial 16S rRNA gene^31,32^. Overall, in other studies investigating head and neck SCC patients using 16S rRNA gene sequencing, the results are not comparable due to the use of different methodological approaches, and sample sizes have been small^19,20^. Our study was conceptualized as a preliminary study and was also limited in sample size. Furthermore, additional medication was applied (analgesic, antibiotic, and antimycotic effects) in accordance with clinical and laboratory findings and guidelines, but differed between individual patients, following the principles of personalized medical treatments. However, we applied standardized sampling based on the Salivette Cortisol kit^12^ and microbiome analysis procedures based on widely used amplification protocols and bioinformatics pipelines. This allowed us to put our data into context with our previous study, in which we described the salivary microbiome of 11 patients with oral and oropharyngeal SCC and compared it to the microbial profile of healthy controls. We observed that changes in the microbial profile mirrored disease progression and reflected clinical preconditions (age, alcohol, tumor size, lymph node status, smoking habit, HPV-positivity)^12^. Including further datasets from our present study, we confirmed our findings regarding the comparison of our primary SCC patients and healthy controls. An increased relative abundance of *Bifidobacterium* was observed in our patient group, whereas the taxon Pasteurellaceae was decreased. Importantly, the fungal microbiome was addressed in the present study and showed an increased relative abundance of fungal signatures, whereas bacterial signatures decreased between healthy and non-treated patients.

Although certain aspects of the microbiome profiles of healthy subjects and SCC patients are different, studies have failed to delineate clear microbial patterns and are partially contradictory. In a previous study based on checkerboard DNA-DNA hybridization of samples from 45 patients and 229 healthy controls, increased counts of *Capnocytophaga gingivalis*, *Prevotella melaninogenica,* and *Streptococcus mitis*^13^ were identified as potential biomarkers for SCCs with a relatively high sensitivity and specificity (both > 80%). Gong et al. focused on the tissue-associated microbiome and identified 26 genera that were significantly different between patients with laryngeal SCC and controls. The highest abundance was observed for *Fusobacterium*, *Prevotella*, and *Gemella* in the patient group^14^. Other studies have reported a relative intraindividual decrease in *Streptococcus* and *Rothia* species in cancer patients when analyzing samples from various sites in the oral cavity^20^ but, like many others, failed to clearly segregate cancer from non-cancer microbiome samples^33^. This indicates a high level of individuality across the microbiome profile and highlights the importance of additional studies with larger cohorts.

In the present study, the impact of RCHT was clearly be observed in the salivary microbiome of SCC patients before and after therapy. We observed changes in the microbial profiles for bacteria and fungi, but not in the archaeal profile due to a substantial number of archaea-negative samples. An association of *Veillonella* and *Actinomyces* signatures with higher cumulative doses (30 to 60 Gy) was reported by Hu et al.^19^, who performed a pyrosequencing-based microbiome analysis of plaque samples taken from subjects undergoing head-and-neck radiotherapy. In our study, however, the relative abundance of *Veillonella* signatures decreased with RCHT. This difference in the abundance of *Veillonella* may be due to the use of different methodological approaches (e.g., sample site, sequencing methods) or the therapy the patients received (e.g., antibiotics, chemoradiation).

In general, microbial changes before and after RCHT may be explained by therapy-associated effects on highly proliferative (tumor), but also healthy proliferative tissue, including the mucosa of the upper respiratory tract. Endotoxins are released, penetrate the submucosa, and induce chemotactic activity on polymorphonuclear neutrophils and macrophages, leading to the activation of pro-inflammatory cytokines and progressive tissue destruction^7,16^. Thus, microbial changes can be explained by the application of RCHT in our cohort.

Microbial dysbiosis and infection can be avoided and treated with antibiotic and/or antimycotic treatment, which was applied to a number of patients in our study. Notably, our data do not indicate a significant impact of antibiotic or, specifically, antimycotic treatment on the microbial changes triggered by RCHT.

The main limitations of our study were the limited sample size and the heterogeneity of the subjects with respect to clinical parameters (medical treatment) and demographics. In addition, the level of alcohol consumption could not be assessed reliably, and the carcinogenic effects cannot be delineated. Furthermore, additional parameters, such as oral hygiene, which may also contribute to oral health and changes in microbial composition, should be considered in future studies.

In this preliminary study, we defined microbial changes in patients before and after RCHT. As probiotic medication has been reported to have a potential protective effect against radiation in the epithelium, future studies should identify the effect of probiotic medication on the oropharyngeal micro- as well as mycobiome in SCC patients undergoing RCHT^34^.

We emphasize the need for larger cohort studies in order to further explore the impact of disease and treatment on the microbiome. Our study should serve as a baseline for further study and be extended towards metagenome-based microbiome analysis to reveal functional and mechanistic information that can be used for improved diagnosis and treatment of patients.

## Materials and methods

### Ethics

The Department of Otorhinolaryngology, University Hospital of Graz, recruited the healthy volunteers and SCC patients. The study design was approved by the Ethics Committee of the Medical University of Graz (EK-Nr. 1325/2015). Ethics approvals were obtained according to the guidelines of the Declaration of Helsinki on biomedical research involving human subjects. Prior to inclusion, all participants provided written informed consent.

### Study subjects

The study included salivary samples from 11 healthy volunteers as controls (10 males, 1 female; mean age 47.8 years, standard deviation 15.1 years) and 31 patients diagnosed with SCC of the head and neck (26 males, 5 females; mean age 60 years, standard deviation 7.5 years). Eleven patients (10 males, 1 female; mean age 58 years, standard deviation 7.6 years) provided samples before and close to the end point of the standard therapy (“after”).

The clinical parameters for all participants are provided in Supplementary Table 1. Information about antibiotic and antimycotic treatments are given in Supplementary Table 2.

### SCC therapy

Recommendations regarding SSC therapy was given by the local interdisciplinary head and neck cancer board. All 11 patients investigated before and after tumor-specific treatment received radiotherapy in combination with platinum-based chemotherapy (10 cisplatin, 1 carboplatin). Radiation doses on the primary tumor side were between 60 and 70 Gy, whereas the radiation dosage in the lymphatic pathways was 50 Gy. Antibiotic and/or antimycotic treatment was applied based on clinical symptoms, local signs of infection, and laboratory findings. Eight of eleven patients received antimycotic treatment (see Supplementary Table S2 and Supplementary Fig. S3).

### Sampling procedure

The Salivette Cortisol saliva detection kit (Sarstedt, Newton, NC, USA) was used to collect samples from all subjects. To stimulate and collect saliva, participants were asked to chew on a provided polyester swab for 60 s before placing it back into the tube. The Salivette Cortisol kit turned out to be a user-friendly method of collecting saliva from both the oral cavity and oropharynx that we also applied in our previous study.

The soaked swab was centrifuged for 5 min at 3000 rpm to obtain clear saliva. The sample was then immediately stored at − 70 °C until further use.

The first collection of patients’ saliva took place after the primary diagnosis of SCC and prior to any specific treatment (chemoradiation therapy, surgery, antibiotics, mycostatin), whereas the second sample was collected after these treatments.

### DNA extraction, PCR, and amplicon sequencing

A MagNA Lyser instrument and MagNA pure LC DNA Isolation Kit III (Roche Diagnostics, Mannheim, Germany) were used to extract microbial DNA according to the manufacturer’s instructions. The microbial 16S rRNA gene was amplified using the universal primer pair 515FB and 806RB for bacterial signatures^35^. A nested PCR approach was used to amplify archaeal signatures. As a first step, the primer pair 344F and 1041R was used, followed by the primer pair 519F and 806R for archaeal signatures^35^. The ITS region was amplified using the ITS86F and ITS4R primer pair to determine fungal signatures^36,37^. Primer sequences and PCR cycling conditions are listed in Supplementary Tables S3 and S4. Sequencing was performed by the Center for Medical Research (ZMF) at the Medical University of Graz using Illumina MiSeq.

### Sequence processing

Raw reads were processed using QIIME2. Briefly, reads were quality filtered and checked for the presence of chimeras before being grouped into RSVs at the level of 99%. To classify the reads, the SILVA 128 database for bacterial^38^, SILVA 132 database for archaeal^38^, and UNITE V8 database for fungal signatures^39^ were used. Extraction blanks (negative controls) were processed in parallel and contaminations due to sample processing were removed using R studio version 1.2.1335 with the R packages phyloseq, ggplot2 and decontam with the threshold set to 0.5 (https://github.com/benjjneb/decontam)^40,41^. Chloroplast signatures and unassigned sequences were also removed. Signatures remaining from the RSV table (Supplementary Datasets 1, 2, and 3) were processed in Calypso^42^. After normalization of the dataset via total sum normalization (TSS), Calypso was used to calculate the Shannon Index (alpha diversity, to visualize evenness and abundance; RSV level), redundancy analysis (beta diversity, to summarize variations in the data set; RSV level), PCoA plot (beta diversity; RSV level), bar chart (relative abundances of different taxa; genus level), bubble plot (relative abundance of different taxa; genus level), LEfSe, to find features that most likely explain differences between groups; genus level), and ANOVA taxa abundance plots (differences in relative abundance of taxa between compared groups; genus level)^43^.

Raw sequence reads are publicly available at the European Nucleotide Archive (PRJEB37299).

### Exclusion criteria

Participants were not allowed to take antibiotics (local or systemic) for at least 4 weeks or receive vaccinations in the 6 months prior to the first sampling. Subjects with long-term antibiotic usage were also excluded.

## Supplementary information

Supplementary file1

Supplementary file2

Supplementary file3

Supplementary file4

Supplementary file5
