# Supplementary material for: Preliminary insights into the impact of primary radiochemotherapy on the salivary microbiome in head and neck squamous cell carcinoma
Source: Sci Rep. 2020 Oct 6;10:16582. doi: 10.1038/s41598-020-73515-0 (PMC7538973; doi:10.1038/s41598-020-73515-0)
Supplement: Supplementary file 4 — Supplementary file4 [file 41598_2020_73515_MOESM4_ESM.pdf]

The impact of primary radiochemotherapy on the salivary microbiome in head and neck squamous cell carcinoma patients: A pilot study.

Christina Kumpitsch[1], Christine Moissl-Eichinger[1,2], Jakob Pock[3], Dietmar Thurnher[3] and Axel Wolf[3]*

[1] Diagnostic and Research Institute of Hygiene, Microbiology and Environmental Medicine, Medical University of Graz, Austria

[2] BioTechMed, Graz, Austria

[3] Department of Otorhinolaryngology, Medical University of Graz, Austria

Supplementary Figure 1: Bubble plot of the 35 most abundant bacterial genera in saliva samples of healthy controls and cancer patients ('diseased').

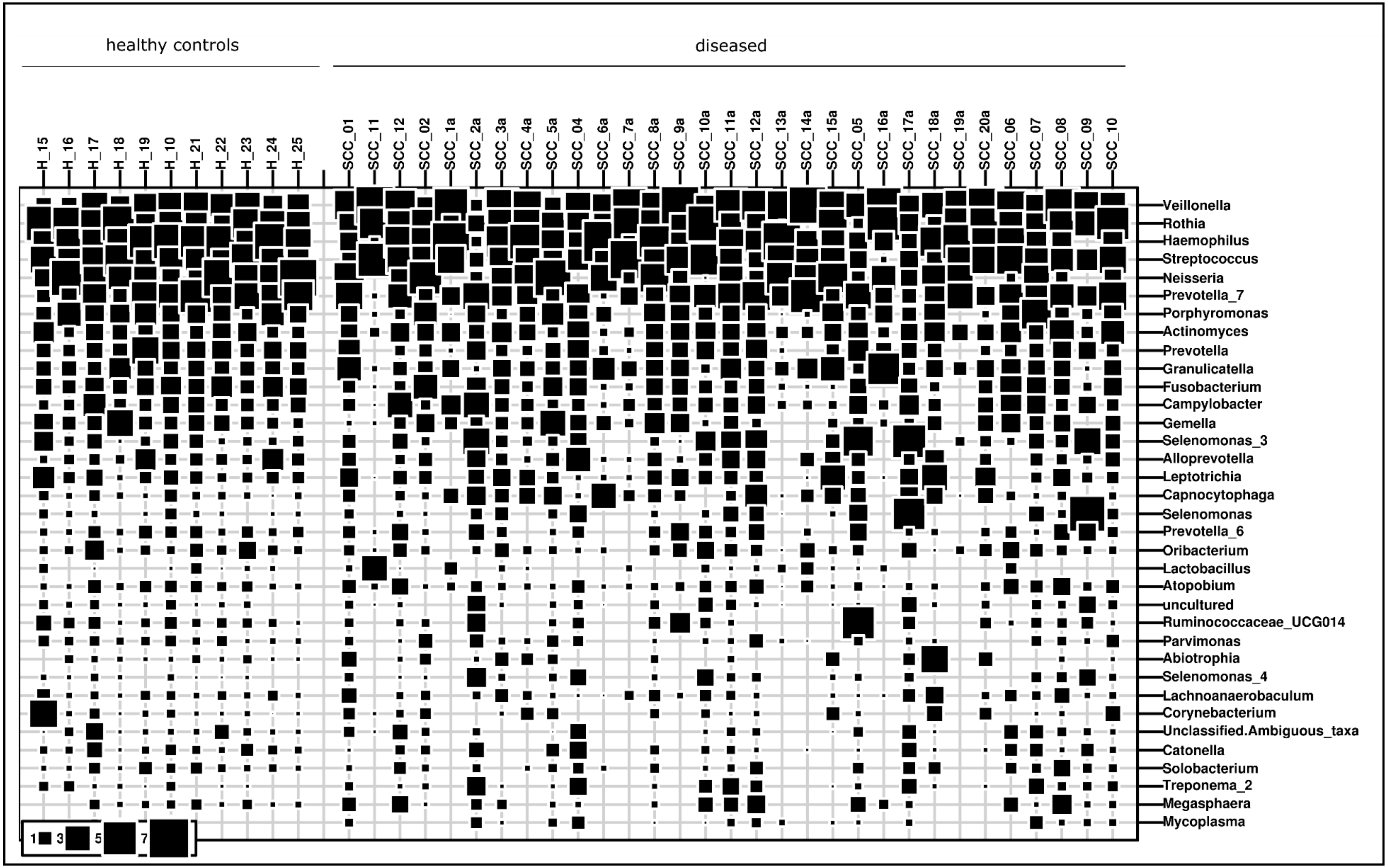

Supplementary Figure 2: Bar chart of the top 35 most abundant bacterial genera in healthy controls and SCC patients ('diseased').

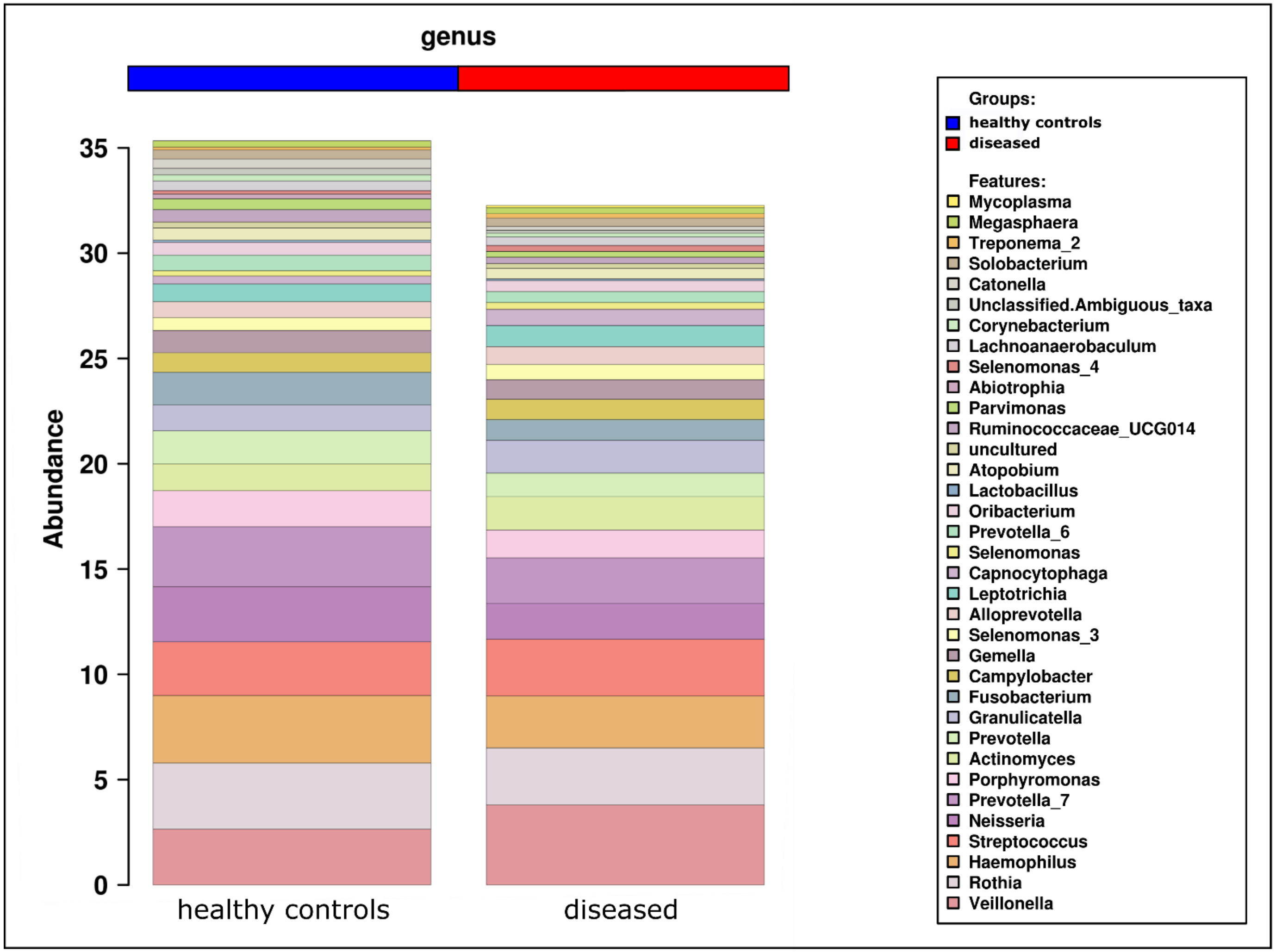

Supplementary Figure 3: Schemata of therapies between the two samplings before and after therapy of 11 SCC patients. X marks the samplings. Duration of time between samplings, and specific therapy in days are noted in brackets.

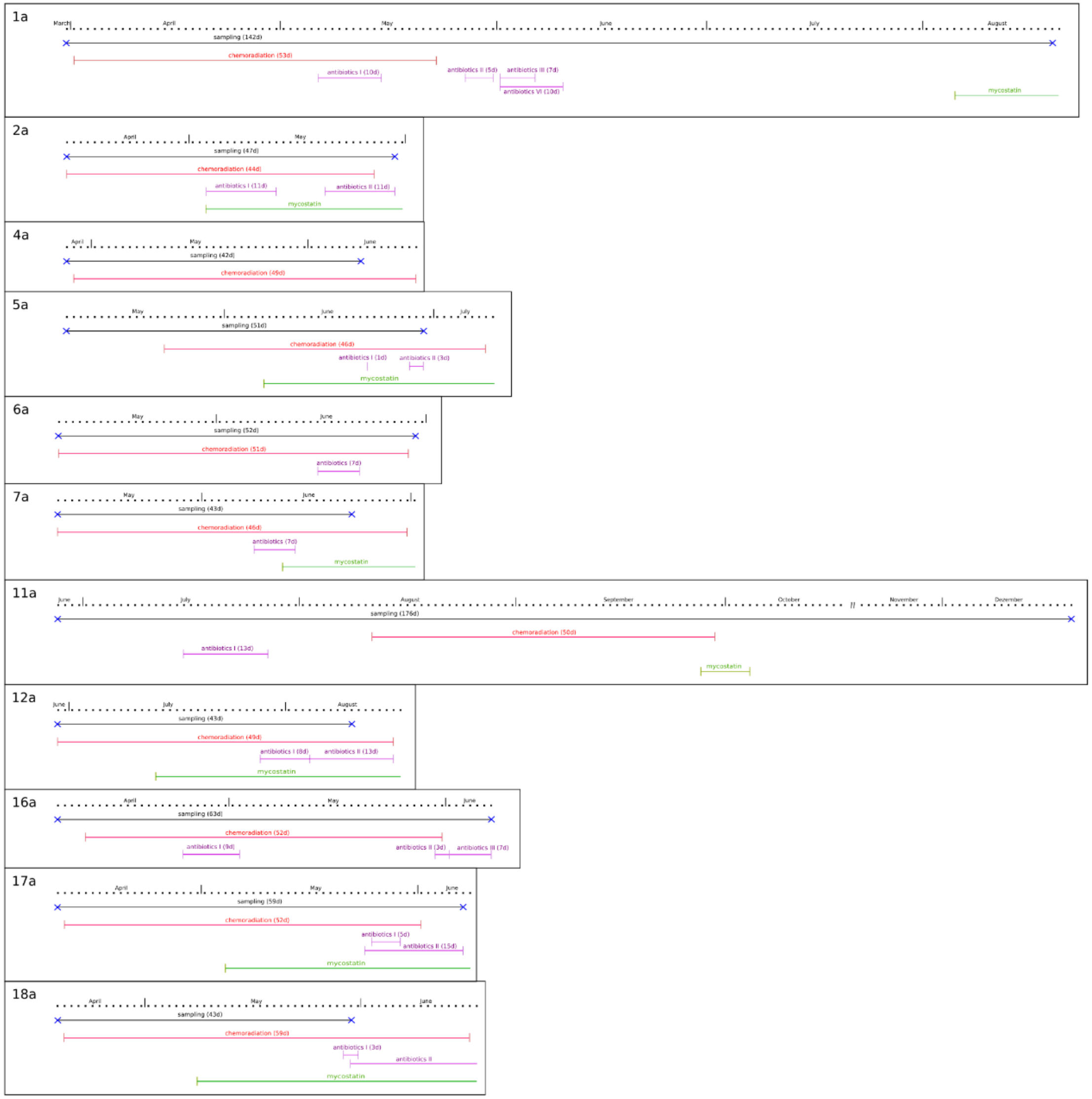

Supplementary Figure 4: PCoA plot of 11 patients before and after therapy at RSV level. Bacterial community grouped differently.

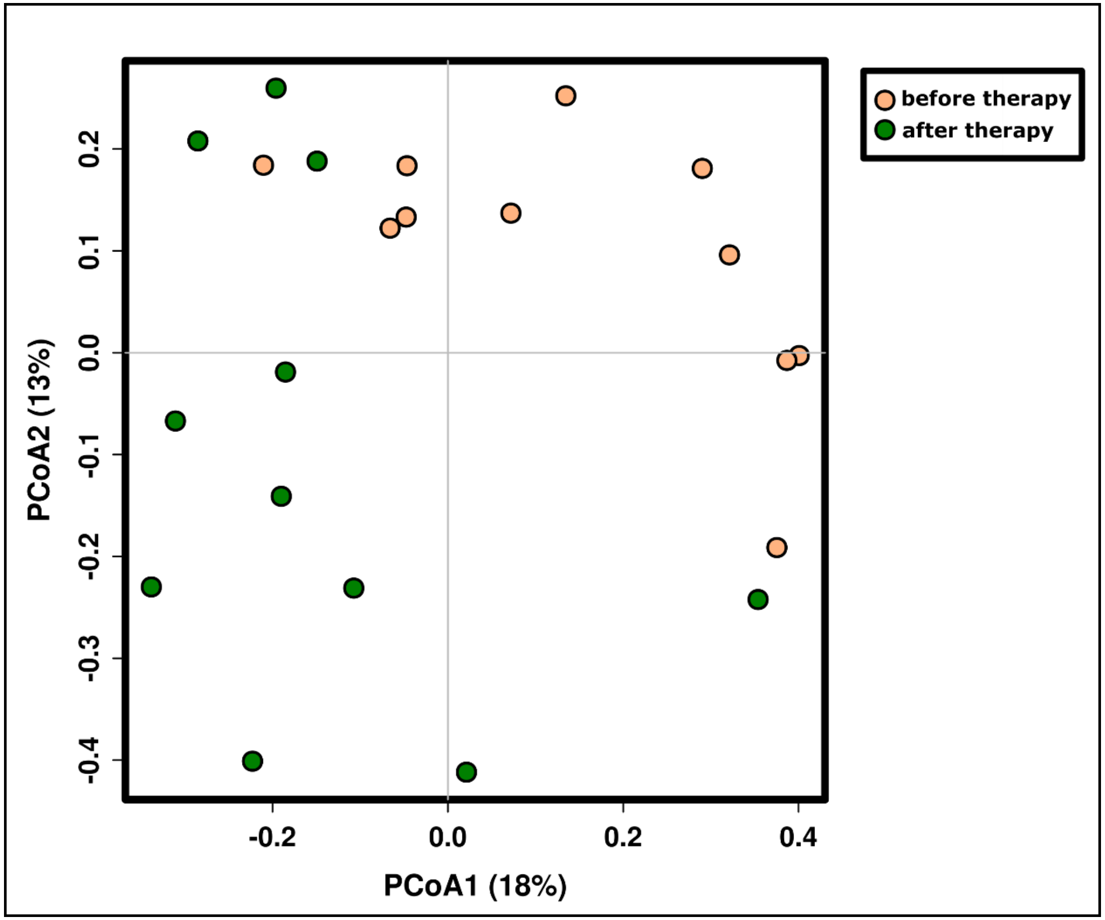

Supplementary Figure 5: RDA+ plot of bacterial community of 11 patients before and after therapy. The analysis showed a significant influence of treatment, smoker and sex on the bacterial profile.

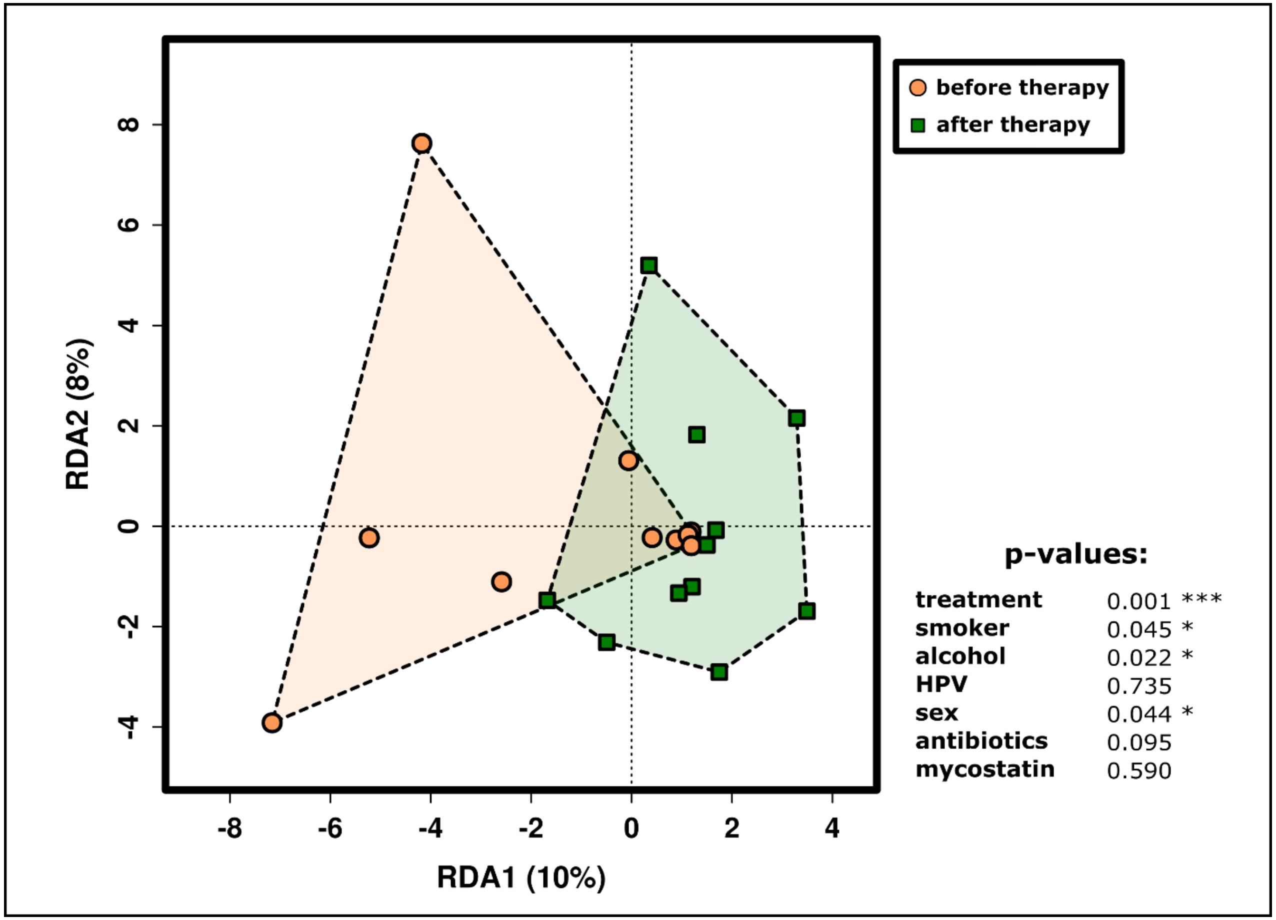

Supplementary Figure 6: Bar chart of the top 35 most abundant fungal genera detected in healthy controls and SCC patients ('diseased').

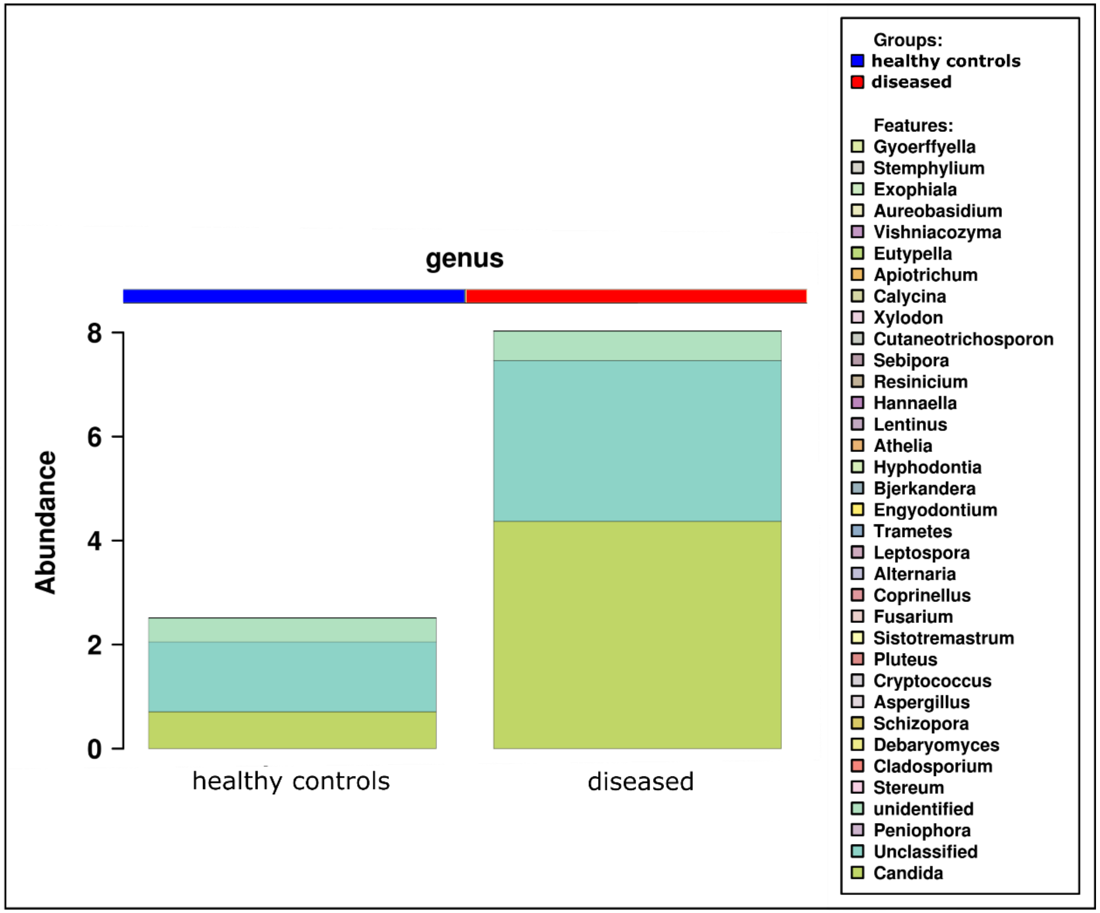

Supplementary Figure 7: RDA plot of patients after therapy (n=11) that were either mycostatin treated or untreated (ANOVA).

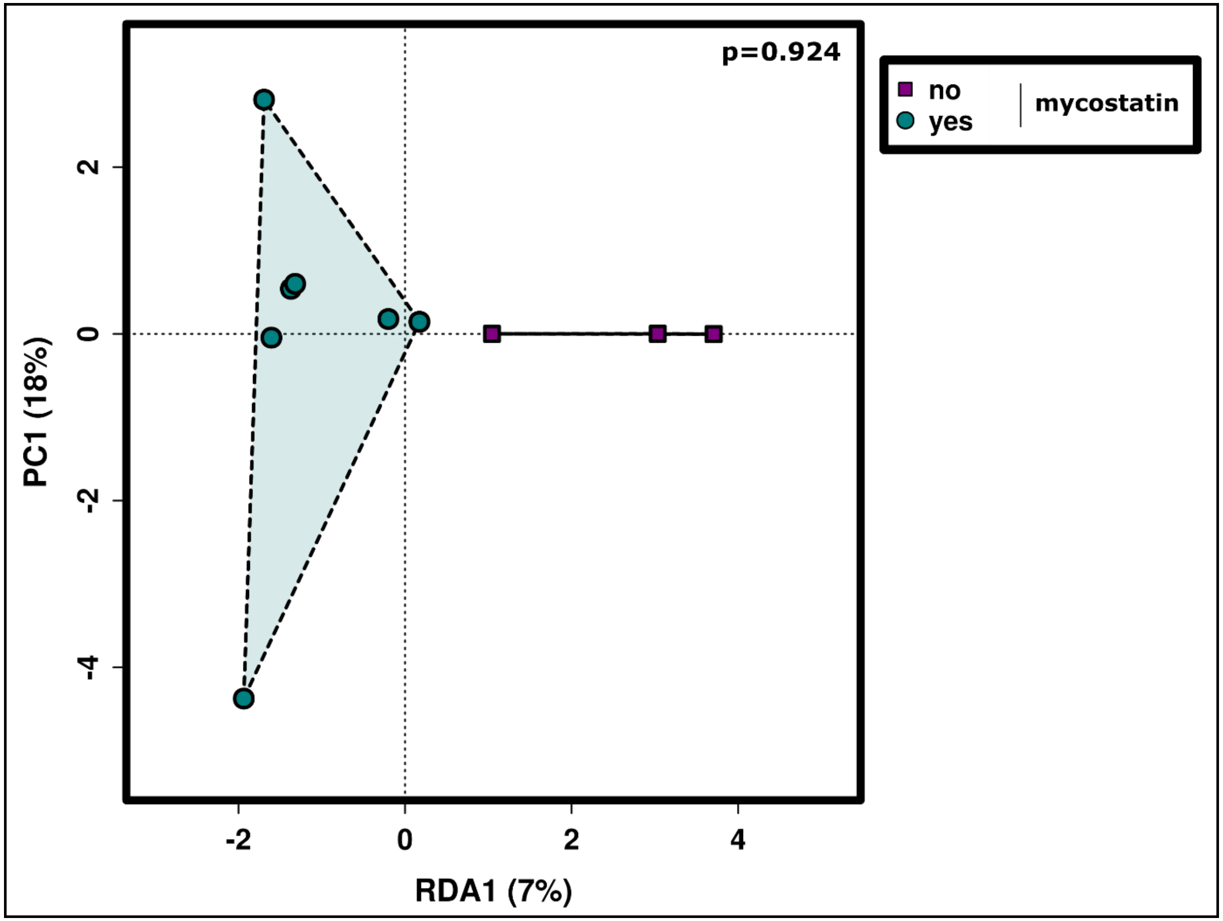

Supplementary Figure 8: a) Bar plot of the top 35 most abundant fungal genera and b) relative abundance (ANOVA) of Candida found in patients after mycostatin therapy (n=11).

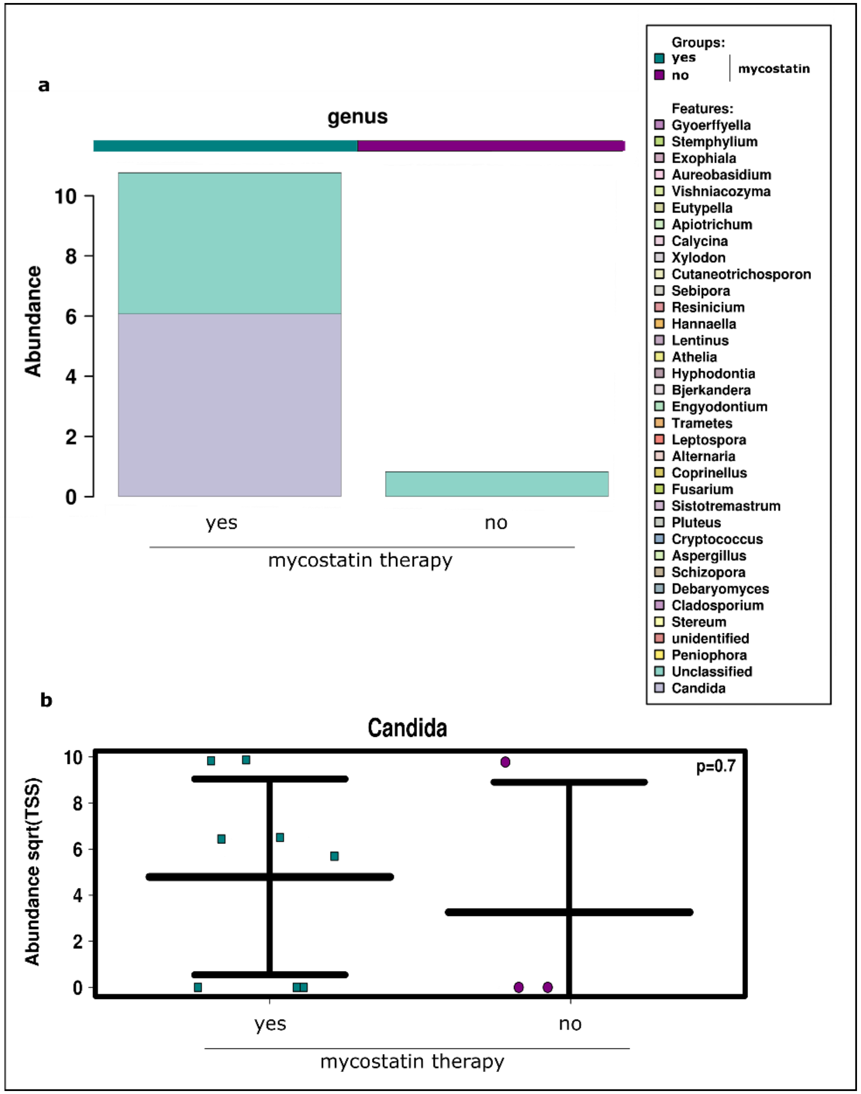

Supplementary Table 2: Therapies of SCC patients who provided samples before and after the standard therapy. (RCHT=radio chemotherapy)

**Participant**	**Duration between samplings**	**Chemoradiation**		**Antibiotic treatment**			**Antimycotic treatment**	
	**days**	**therapy**	**duration (days)**	**therapy**	**duration (days)**		**therapy**	**duration (days)**
**1**	141	RCHT+cisplatinum	52	moxifloxacin	9		nystatin	14+
				amoxicillin/clavulanacid	4			
				piperacillin/ tazobactam	5			
				moxifloxacin	9			
**2**	47	RCHT+cisplatinum	44	amoxicillin/clavulanacid	10		nystatin	27+
				amoxicillin/clavulanacid	10			
**4**	42	RCHT+cisplatinum	49	-	0		-	-
**5**	51	RCHT+cisplatinum	46	cefuroxim	n.a.		nystatin	26+
				amoxicillin/clavulanacid	2			
**6**	51	RCHT+cisplatinum	50	amoxicillin/clavulanacid	6		-	-
**7**	42	RCHT+cisplatinum	50	ciproxin	6		nystatin	10+
**11**	175	RCHT+cisplatinum	49	piperacillin/ tazobactam	12		nystatin	7
**12**	42	RCHT+cisplatinum	48	amoxicillin/clavulanacid	7		nystatin	28+
				moxifloxacin	12			
**16**	62	RCHT+cisplatinum	51	amoxicillin/clavulanacid	8		-	-
				amoxicillin/clavulanacid	2			
				moxifloxacin	6			
**17**	58	RCHT+cisplatinum	51	amoxicillin/clavulanacid	4		nystatin	34+
				amoxicillin/clavulanacid	14			
**18**	42	RCHT+cisplatinum	58	moxifloxacin	2		nystatin	22+
				piperacillin/ tazobactam i.v.	n.a.			

Supplementary table 3. Primer pairs for amplicon sequencing. *Primer names according to [33]

PCR approach	Primer name	direction	Sequence (5′– >3′)	Reference
universal	515FB*	forward	TCGTCGGCAGCGTCAGATGTGTATAAGAGACAG**GTGYCAGCMGCCGCGGTAA**	Pausan et al., 2019 [33]
	806RB*	reverse	GTCTCGTGGGCTCGGAGATGTGTATAAGAGACAG**GGACTACNVGGGTWTCTAAT**	Pausan et al., 2019 [33]
archaea (nested PCR 1)	344F*	forward	ACGGGGYGCAGCAGGCGCGA	Pausan et al., 2019 [33]
	1041R*	reverse	GGCCATGCACCWCCTCTC	Pausan et al., 2019 [33]
archaea (nested PCR 2)	519F*	forward	TCGTCGGCAGCGTCAGATGTGTATAAGAGACAG**CAGCMGCCGCGGTAA**	Pausan et al., 2019 [33]
	806R*	reverse	GTCTCGTGGGCTCGGAGATGTGTATAAGAGACAG**GGACTACVSGGGTATCTAAT**	Pausan et al., 2019 [33]
fungi	ITS86F	forward	TCGTCGGCAGCGTCAGATGTGTATAAGAGACAG**GTGAATCATCGAATCTTTGAA**	Turenne et al., 1999 [34]
	ITS4R	reverse	GTCTCGTGGGCTCGGAGATGTGTATAAGAGACAG**TCCTCCGCTTATTGATATGC**	White et al., 1990 [35]

Supplementary table 4. PCR conditions for different 16S rRNA sequencing approaches.

	Universal	Archaea		Fungi	
		(nested PCR 1)	(nested PCR 2)	(step1-4)	(step5-8)
**Initial duration**	94℃, 3‘	95℃, 5‘	95℃, 5‘	95℃, 5‘	
**Denaturation**	94℃, 45‘‘	94℃, 30‘‘	95℃, 40‘‘	95℃, 30‘‘	95℃, 30‘‘
**Annealing**	50℃, 60‘‘	56℃, 45‘‘	63℃, 2‘	56℃, 30‘‘	52℃, 30‘‘
**Elongation**	72℃, 1‘30‘‘	72℃, 1‘	72℃, 1‘	72℃, 1‘	72℃, 1‘
**Final elongation**	72℃, 10‘	72℃, 10‘	72℃, 10‘		72℃, 10‘
**No. of cycles**	35	25	30	20 (step2-4)	15 (step 5-7)
